# The Swiss national strategy for spinal cord injury as a model of inclusive public health policy

**DOI:** 10.3389/fresc.2026.1878464

**Published:** 2026-10-21

**Authors:** Sara Rubinelli, Gerold Stucki, Mirjam Brach, Heidi Hanselmann, Michael Jordi, Claudia Zanini

**Affiliations:** 1 Department of Health Sciences and Medicine, University of Lucerne, Lucerne, Switzerland; 2 Swiss Paraplegic Research, Nottwil, Switzerland; 3 Swiss Paraplegic Foundationm, Nottwil, Switzerland; 4 Independent counsellor, Bern, Switzerland

**Keywords:** disability, equity, learning health system, paraplegia, rehabilitation, stakeholder involvement

## Abstract

**Background:**

Spinal cord injury (SCI) is a condition requiring lifelong coordinated health and social services. Despite high-quality rehabilitation in Switzerland, gaps persist in long-term follow-up, psychosocial support, and equitable access to services across regions. This article describes the development of the Swiss National Strategy for SCI (2025–2033) and its relevance for public health policy.

**Methods:**

The strategy was developed through a structured participatory process combining scientific evidence, stakeholder consultation, and multi-level governance. Findings from the Swiss Spinal Cord Injury Cohort Study (SwiSCI) and related studies informed the identification of needs and system gaps. People with SCI, caregivers, clinicians, researchers, insurers, NGOs, and public authorities contributed through consultation rounds and stakeholder workshops to identify and prioritize strategic objectives.

**Results:**

Nine fields of action were identified, including safeguarding existing services, prevention, lifelong follow-up, psychosocial support, accessibility, employment, caregiver support, and data-driven quality development. Inclusion was defined as a cross-cutting principle integrated across all fields through measures addressing barriers, strengthening capabilities, and supporting participation. Each field includes specific goals and measures. Early implementation follows annual prioritization cycles, with selected measures further developed through dedicated working groups.

**Conclusions:**

This article shows that a national strategy can originate through a participatory, bottom-up approach and translate shared priorities into a governance framework designed to support implementation. It may inform similar efforts in other countries.

## Introduction

Spinal cord injury (SCI) is a lifelong health condition with far-reaching implications for functioning, participation, and overall well-being (1, 2). Beyond the acute phase, SCI requires coordinated, long-term management across medical, rehabilitative, psychological, and social domains. For individuals, it often represents a profound change in independence and identity; for health systems, it exposes the capacity to provide sustained, equitable, and person-centered support (3, 4). SCI, therefore, serves as a strategic case for examining the strengths and weaknesses of health and social care systems in countries.

The World Health Organization's International Perspectives on Spinal Cord Injury (IPSCI) report presented SCI as an illustrative case to demonstrate how rehabilitation can optimize functioning across the life course. The Swiss National Strategy for SCI can be seen as a national-level implementation of the IPSCI vision, translating its principles into concrete action.

Switzerland is internationally recognized for its high-quality SCI rehabilitation. The system is characterized by highly specialized acute rehabilitation in four centers, combined with outpatient follow-up and community-based services (5). Core components include interdisciplinary rehabilitation, long-term medical follow-up, and access to assistive technologies (e.g., mobility aids, transfer aids and environmental control systems provided through disability insurance) and home adaptations (e.g., Centre for Obstacle-Free Building) (6–8). Financing is organized through a combination of accident and health insurance schemes, which differ in scope and may affect continuity of support over the life course (9).

A further structural feature of the Swiss SCI context is the integration of research, clinical, and community-based actors through established data infrastructures. The Swiss Spinal Cord Injury Cohort Study (SwiSCI) (10, 11) provides longitudinal national data on functioning, health, and quality of life, while the International Spinal Cord Injury (InSCI) (12) survey enables cross-national comparison. Together, these infrastructures support both the development of the national strategy and the monitoring of its implementation over time.

Despite this well-developed system, providing equitable and consistent access to long-term follow-up remains a challenge. Evidence highlights gaps in psychosocial support, continuity of care, and access to community services, with disparities mainly affecting women, older adults, and people in rural areas (13–19). These inequalities occur amid demographic and systemic pressures: an aging population with SCI, a higher rate of injuries among older individuals, increasing complexity of multimorbidity, workforce shortages, financial limitations, and constraints in rehabilitation length of stays (20–27).

In response, a coordinated action between Swiss Association of SCI Centers, Swiss Paraplegic Foundation, Swiss Paraplegic Association and Swiss Paraplegic Research has developed the National Strategy for SCI (2025–2033), a national roadmap designed to strengthen long-term, person-centered SCI care. The strategy was launched on September 8, 2025, in Berne and is available in both a full and a shorter version (28).

This paper describes the development and early implementation phase of the Swiss National Strategy for Spinal Cord Injury. It documents how a national rehabilitation strategy was developed through evidence synthesis, stakeholder engagement, and multi-level governance in a decentralized health system.

While focused on SCI, the process and governance approach may be relevant for other populations requiring rehabilitation and long-term support, including people living with stroke, traumatic brain injury, musculoskeletal conditions, or chronic neurological diseases. SCI is used as an illustrative case to examine how rehabilitation priorities can be structured within complex systems with multiple actors and responsibilities.

At the international level, the strategy contributes to ongoing discussions on strengthening rehabilitation systems. It aligns with WHO's Rehabilitation 2,030 initiative (29), supports Resolution WHA76.6 (30), and reflects the principles of the UN Convention on the Rights of Persons with Disabilities (31).

## Methods

### Conceptual framework

The Swiss National Strategy for SCI is grounded in a conceptual foundation drawing on health-systems thinking, rehabilitation science, participatory design, and an ethical commitment to equity and inclusion (32). Building on this foundation, the strategy focuses on real-world factors that affect functioning across the life course (33, 34). Rather than organizing priorities around clinical specialties, institutional mandates, or phases of care, the strategy structures its action fields around key domains influencing functioning, including access to specialized rehabilitation and lifelong follow-up, prevention and management of health conditions, psychosocial support, accessibility, participation, and the social and policy context shaping autonomy and daily living. This approach ensures that the strategy reflects the complexity of real-world life with SCI and that its interventions address healthcare as well as the broader ecosystem that enables people to live well.

### Strategy development process

The Swiss National Strategy for SCI was developed through a structured participatory process combining scientific evidence with stakeholder deliberation. This process involved people with SCI alongside clinicians, researchers, policymakers, and insurers (35, 36). Participants were identified through an iterative stakeholder mapping process based on an initial core group and expanded through professional networks and recommendations.

#### Evidence synthesis

The strategy development started with a focused review of key scientific evidence on SCI in Switzerland. This review mainly used publications based on data from the Swiss Spinal Cord Injury Cohort Study (SwiSCI) (10) and the International Spinal Cord Injury Community Survey (InSCI) (37). To contextualize this evidence, the review was supplemented by informal consultations with clinicians, researchers, and professionals active in SCI care and policy. These exchanges provided practical insights into how the system currently responds to the needs of people with SCI and where persistent challenges remain.

#### Governance structure

A multi-level governance structure was established to support stakeholder involvement throughout the strategy development process. A national kick-off meeting was held in June 2023 to present the strategic vision, gather input, and identify key actors for long-term involvement. Based on feedback from this meeting, the governance structure was further developed and refined to support broader stakeholder involvement in the strategy development process.

#### Stakeholder consultations

Two formal consultation rounds with the Expert Panel (5 members) and the SCI Advisory Board (9 members) were conducted in May and September 2024. In preparation for the first round, participants received a consultation package including a list of the proposed fields of action, associated measures, and a summary of key scientific findings. The full consultation package (in German) is available from the corresponding author upon request. Participants completed an online assessment, in which they rated each proposed measure by priority level (high, medium, low) and provided written justifications. The three response categories formed an ordinal priority scale. No predefined numerical threshold was used to define consensus. Given the small size of the two groups, the ratings were used to identify majorities. Measures receiving a majority of high-priority ratings in both groups were retained without further discussion, while measures receiving a majority of low-priority ratings in both groups were removed. The ratings were also used to identify measures requiring further discussion. Subsequent in-person meetings focused on these measures and used the written justifications to initiate the discussion. Discussions resulted in the confirmation, removal, reformulation, or splitting of proposed measures. Participants could also propose additional measures which were incorporated when supported by the group. Feedback from the Expert Panel was synthesized and presented to the SCI Advisory Board, ensuring that the second round built directly on prior input.

#### Prioritization and consolidation

Following the consultation rounds, the Steering Committee met on 6 November 2024 to validate the revised list of measures, based on synthesized input from both rounds of consultation with expert bodies. Subsequently, a national stakeholder workshop was held with 35 participants. Participants were divided into four working groups and prioritized the strategic objectives using predefined criteria such as impact, feasibility, urgency, and system alignment. Each working group was assigned two fields of action and tasked with reviewing the corresponding strategic aims. Each group was facilitated by a moderator and supported by a note taker.

The main reduction in the number of measures occurred at this stage: aims receiving lower priority were not retained, and the measures associated with these aims were consequently removed. The Coordination Office systematically summarized the notes to inform prioritization decisions. The final set of measures was then formally approved by the Steering Committee in February 2025.

A preliminary version of the strategy was then drafted and submitted for review to the Steering Committee in April 2025. Feedback from this session informed the final refinements, ensuring that the strategy was aligned with the perspectives of political, institutional, clinical, and lived-experience stakeholders. The timeline of the whole process is presented in Figure 1.

**Figure 1:**
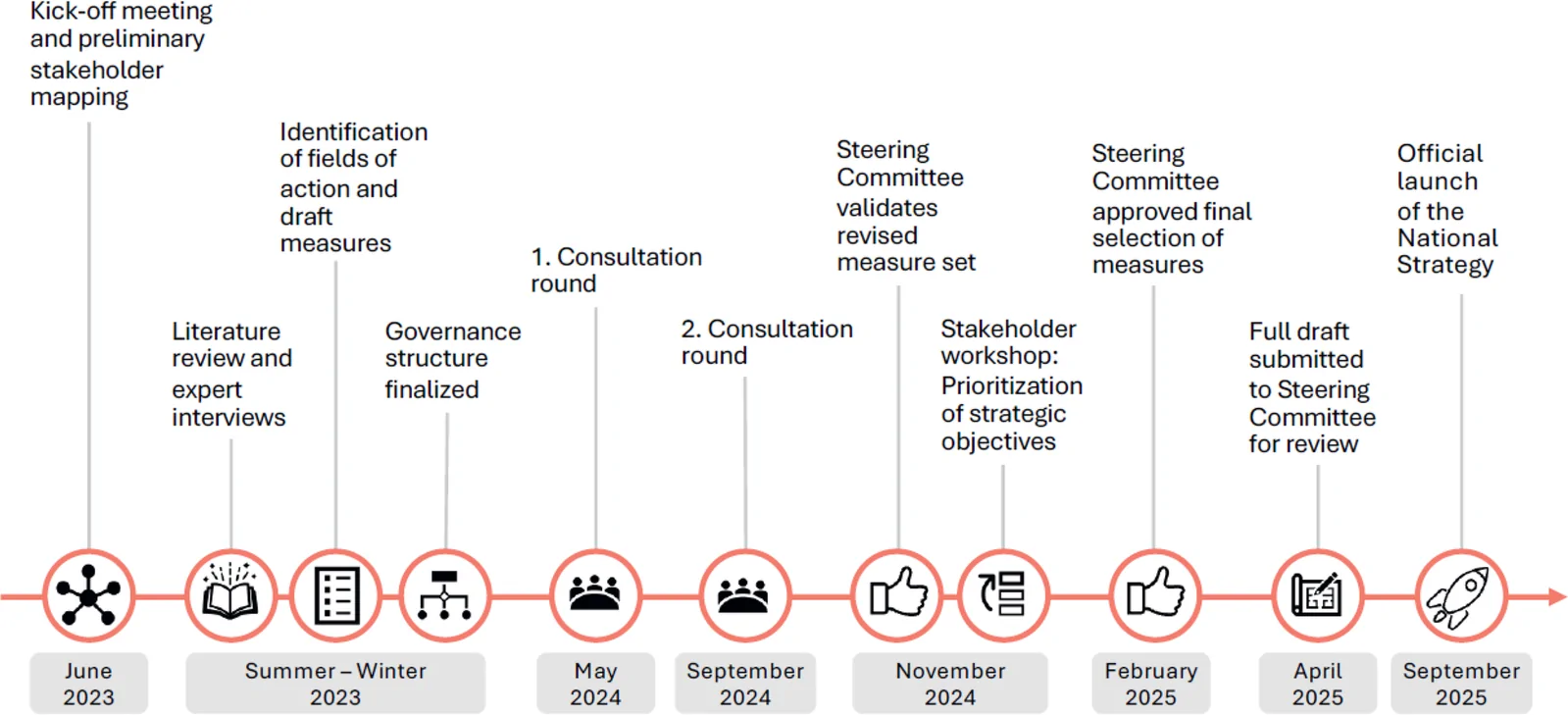
Timeline and milestones.

### Data analysis

Quantitative data from the online assessments were analyzed descriptively, based on the distribution of priority ratings (high, medium, low) across the Expert Panel and the SCI Advisory Board. Written justifications, open-text inputs, and workshop notes were synthesized by the Coordination Office: inputs were grouped by field of action, recurring arguments were summarized, and synthesis documents were prepared to support deliberation and decision-making in stakeholder meetings and Steering Committee sessions. No formal qualitative analytic framework was applied.

### Early implementation phase

Implementation of the Swiss National Strategy for SCI is supported by a multi-level governance structure. Strategic oversight is provided by a Steering Committee including representatives from SCI centers, national SCI organizations, disability advocacy groups, and accident insurers. Federal and cantonal authorities participate as observers.

A Coordination Office ensures linkage between strategic decisions and operational implementation (0.6 FTE coordinator position). An Implementation Advisory Group provides input from policy, service delivery, monitoring, and lived experience perspectives.

From 2026 onwards, implementation follows annual prioritization cycles overseen by the Steering Committee. The Coordination Office prepares a list of proposed measures based on predefined criteria (feasibility, expected impact, policy relevance, contribution to inclusion, and sustainability), supported by input from implementation partners. Measures are selected through discussion and agreement within the Steering Committee.

Implementation follows an iterative learning approach (38, 39). Where available, existing indicators are used to monitor progress. For several measures, no dedicated indicator systems are currently in place; in these cases, process-related indicators (e.g., implementation progress, reach, and uptake) are used as primary markers of success, complemented by existing routine data where possible.

### Funding/resources

The development process was primarily supported through existing institutional resources of the participating organizations. No dedicated external funding stream was established for the strategy development, and a formal costing exercise was not conducted during this phase.

## Results

### Governance structure

The initial governance structure consisted of a small Steering Committee, a restricted stakeholder Forum designed to engage actors with an indirect role in SCI care, a three-member Advisory Board of persons with lived experience, and a dedicated Expert Panel (scientific and clinical expertise). Following the June 2023 kick-off meeting, the governance structure evolved to strengthen stakeholder representation.

The Steering Committee was expanded to include people with SCI as full members, and representatives of the Swiss Conference of Cantonal Health Directors (GDK) and the Federal Office of Public Health (FOPH/BAG) were granted observer status, strengthening federal and cantonal alignment. The expanded Steering Committee comprised five full members and two observers.

The stakeholder Forum initially included representatives from key medical, rehabilitation, disability, service provider, insurance, and public sector organizations. During the development process, it was expanded to include additional rehabilitation professions (nursing, physiotherapy, occupational therapy, nutrition, speech and language therapy, psychology, and health-related social work), umbrella organizations for social care providers, health insurance associations, national quality monitoring bodies and national home-care services. The expanded Forum comprised representatives from 18 organizations, broadening representation across the SCI care and support ecosystem.

The Advisory Board of people with lived experience was expanded from three to nine members and included one family caregiver. The Expert Panel, composed of five members, remained unchanged throughout the process.

Figure 2 presents the final governance structure, while Table 1 lists the different stakeholders engaging in the development of the national strategy and their main tasks.

**Figure 2:**
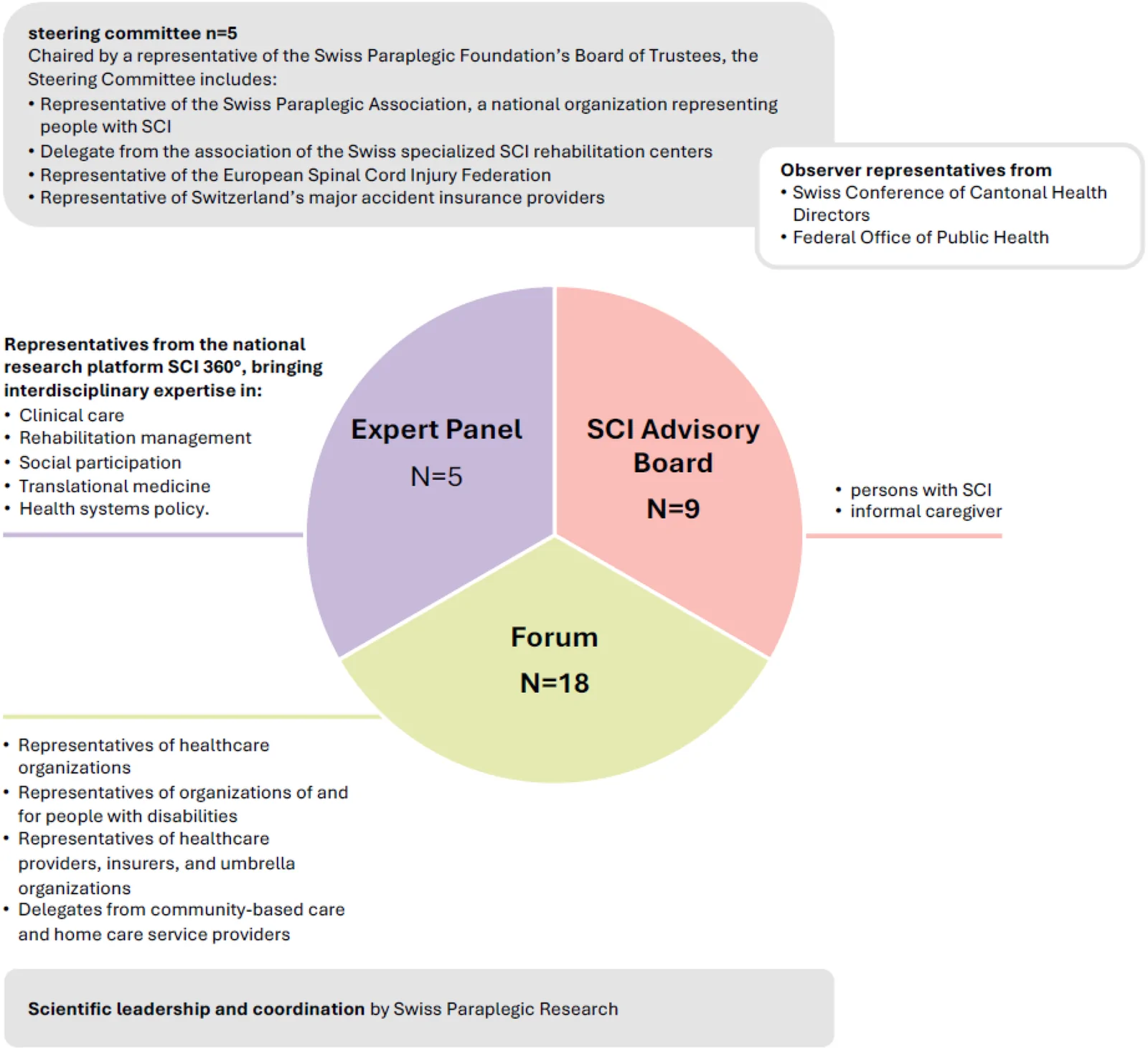
Governance.

**Table 1:** Stakeholders and their role in strategy development.

Stakeholder type	Example entities	Role in strategy development
People living with SCI	–	Co-design of goals and measures; feedback on relevance; ongoing governance input
Informal caregivers	–	Input on long-term care needs and system gaps, especially around support and navigation
Disability organizations and user organizations	Swiss Paraplegic Association, Wheelchair Clubs, Inclusion Handicap	Advocacy for inclusion and accessibility; lived-experience perspective; support for participatory governance
Medical professionals	Professional associations (e.g., Swiss Society of Physical Medicine and Rehabilitation/Reha Schweiz, FMH)	Identification of gaps in care; prioritization of goals; validation of proposed measures
Rehabilitation and social care professionals	Professional associations representing nursing, physiotherapy, occupational therapy, psychology, social work, vocational rehabilitation, and other relevant professions	Identification of gaps across the rehabilitation pathway; prioritization of goals; validation of proposed measures
Healthcare provider organizations	Swiss Association of SCI Centers, SW!SS REHA (Association of leading rehabilitation clinics in Switzerland), H + (Swiss Hospitals and Clinics Association)	Representation of rehabilitation and hospital providers; contribution of expertise on care organization, service delivery, quality requirements, and feasibility of implementation
Community and home care providers	ParaHelp, Spitex Switzerland	Practical insights on continuity of care and regional implementation needs
Health and accident insurers	prio.swiss, Suva	System-level alignment; financial and regulatory perspectives
Researchers	Swiss Paraplegic Research	Evidence synthesis; methodological design; monitoring perspective
Federal and public health institutions	FOPH, GDK	Alignment with national and cantonal health policy frameworks; system legitimacy and sustainability

### Fields of action and measures

The evidence synthesis informed the identification of eight fields of action in the initial strategy draft, addressing key gaps and priorities in SCI care and participation: prevention of SCI, prevention and management of secondary conditions, follow-up care, mental health, accessibility, employment, caregiver support, and data use. Each field was linked to strategic goals and an initial draft list of measures.

A ninth field of action, 'Safeguard what works,' was added following stakeholder consultation. This addition reflects the importance of explicitly considering existing strengths within the Swiss SCI system alongside identified gaps. Table 2 presents the nine fields of action.

**Table 2:** Fields of action, with their focus, rationale, and examples of proposed measures, and contribution to inclusion.

Action field	Strategic focus	Why it matters	Key measures	Contribution to inclusion
1. Safeguard what works	Sustain excellence in rehabilitation and core services	Existing services are effective but depend on fragile structures and limited long-term investment	Secure sustainable financing models, strengthen academic integration of rehabilitation, invest in interdisciplinary workforce	Improve equitable access to high-quality SCI rehabilitation and long-term support structures
2. Prevention of SCI	Prevent traumatic and age-related SCI	Many SCI cases are preventable, but national efforts remain limited and fragmented	Develop targeted fall prevention for older adults and prevention campaigns for youth, expand community-level initiatives	Supports equitable access to prevention initiatives across population groups and regions
3. Prevention and management of secondary conditions & comorbidities	Support prevention, early detection, monitoring, and management of secondary health conditions and comorbidities	Secondary health conditions and comorbidities (e.g., infections, cardiometabolic problems, musculoskeletal complications) reduce quality of life and autonomy and increase healthcare costs. Screening, personalized care planning, and self-management support can aid early detection and management, but these approaches alone cannot address the full burden of secondary conditions, and further research is needed.	Implement structured screening protocols, personalize care plans, and provide digital self-management tools	Supports autonomy and participation by reducing preventable health limitations
4. Mental health & psychosocial support	Strengthen psychosocial support from rehabilitation through community life	Psychological needs are frequent post-rehabilitation but are not systematically addressed	Expand peer and psychological support during and after rehab, ensure trauma-informed services, and better integrate mental health in follow-up	Addresses barriers to social participation and supports adjustment across the life course
5. Lifelong medical follow-up	Ensure lifelong, regionally accessible, and coordinated care	Follow-up and care are uneven across regions, and older adults or women often face greater access barriers, increasing health risks	Promote telemedicine, strengthen links between primary and specialized care, and implement national hotline	Promotes continuous access to appropriate care regardless of region and changing needs
6. Mobility & accessibility	Promote physical accessibility and equitable mobility	Environmental and financial barriers reduce autonomy and limit participation	Enforce accessibility legislation, improve funding of mobility aids post-retirement, and support home/transport access improvements	Reduces environmental barriers that limit independence and participation
7. Employment & vocational inclusion	Improve labor market participation and retention	Many people return to work after SCI, but maintaining employment over time remains challenging.	Promote person-job matching, provide ongoing job coaching beyond reintegration, promote part-time work models	Supports participation in working life and equal opportunities
8. Informal caregiver support	Strengthen recognition and support for informal caregivers	Family caregivers provide essential care but experience long-term burden with limited support	Offer financial support, structured training, navigation services	Reduces inequalities in access to informal support and helps maintain participation in daily life
9. Data-driven quality development	Enable continuous system improvement through data integration	Current data systems are fragmented; integrated data is essential for evaluating and improving services	Improve the use of routine data, collect data on functioning and comorbidities, establish outcome indicators, build participatory feedback and evaluation systems	Enables identification of disparities and monitoring of equitable outcomes

An initial set of 54 proposed measures was presented during the consultation rounds. Through confirmation, reformulation, splitting, removal, and the addition of measures proposed by the Expert Panel and the SCI Advisory Board, this resulted in 60 proposed measures for subsequent prioritization. The national stakeholder workshop then prioritized the strategic aims, resulting in a consolidated set of 16 aims and 32 associated measures.

Together, the nine fields of action reflect a functioning-centered approach spanning prevention, rehabilitation, long-term support, and participation. Inclusion is considered a cross-cutting principle and is integrated across all action fields through measures aimed at reducing barriers, strengthening capabilities, and supporting equal participation.

The nine fields of action are interrelated and reflect the complexity of life with SCI. Several fields operate in close connection, particularly lifelong follow-up care, prevention and management of secondary conditions, mental health and psychosocial support, accessibility, and employment. For example, strengthening lifelong follow-up care requires not only specialized medical services but also on workforce capacity, digital tools, and assistive technologies. Similarly, participation outcomes require coordination across health, social, and labor systems, as barriers to inclusion often extend beyond healthcare services. Technological developments are addressed within relevant fields of action, including digital self-management tools, telemedicine, assistive technologies, and data infrastructure.

### Early implementation phase

Building on the adopted fields of action, the strategy entered its first annual implementation cycle. Three measures were selected for initial implementation in 2026: respiratory muscle training, psychosocial support for the transition from rehabilitation to everyday life, and informal caregiver support. For each measure, interdisciplinary working groups were established to analyze current practice, refine priorities, and prepare implementation steps.

A detailed example of the respiratory muscle training measure is provided in Box 1.

Box 1 **Example of early implementation process: respiratory muscle training.**

**Table:** 

Based on initial discussions within the working group, three potential priority directions for implementation were identified: (1) defining best practices and areas requiring harmonization across rehabilitation centers, (2) developing systematic monitoring of respiratory muscle function during annual follow-up for higher-risk groups, and (3) integrating indicators of respiratory complications and respiratory muscle training into the SwiSCI Community Survey to enable long-term monitoring of trends. To inform these proposals, a brief survey was conducted across the four specialized rehabilitation clinics to describe current practice in acute rehabilitation and follow-up. The survey found that respiratory muscle training is established in all four clinics and that a shared basic practice already exists. Differences were identified in the standardization of training delivery and in the use of outcome measures. In addition, follow-up and support for home-based training after discharge were often unstructured or absent. These findings provide an empirical basis for further refining the proposed implementation priorities and for discussions on harmonization across centers.

## Discussion

### Main findings and lessons from developing a national SCI strategy

This study describes the development and early implementation of the Swiss National Strategy for SCI. The strategy was developed through a structured participatory process involving clinical, research, policy, insurer, and lived-experience stakeholders, supported by iterative consultations and multi-level governance. The process resulted in the definition of 16 aims and 32 measures across nine fields of action, followed by the initiation of early implementation activities for three priority measures. The experience provides insights into the opportunities and challenges of developing a national strategy in a decentralized health system, particularly regarding stakeholder alignment, governance, and the transition from priority setting to implementation.

The Swiss experience highlights that developing a national rehabilitation strategy requires more than identifying unmet needs or defining priorities. It requires mechanisms that can support the translation of shared priorities into coordinated action across different actors and levels of the health system. In a decentralized and participatory governance setting alignment is achieved through intensive coordination rather than hierarchical authority. This created a tension between inclusiveness and decision speed: broad stakeholder involvement increases legitimacy and ownership, while also requiring substantial effort to synthesize perspectives, clarify differences, and maintain shared understanding across actors.

Within this process, disagreement was often linked to different institutional responsibilities, priorities, or strategic perspectives. Making these differences explicit became an important task of the coordination process, as it allowed stakeholders to clarify positions and identify feasible solutions before formal decisions were taken. Coordination therefore emerged not as an administrative support function but as a central mechanism enabling collective decision-making through continuous communication, iterative synthesis of inputs, and feedback loops.

### Moving from priority setting towards implementation

The participatory orientation of the Swiss strategy is consistent with international efforts to involve people with SCI in defining priorities. In the United Kingdom, a James Lind Alliance priority setting partnership brought together people living with SCI, caregivers, and health professionals to identify the top ten research priorities for SCI (40); a comparable nationwide exercise was conducted in Sweden (41), embedded in a broader needs-assessment project (4). In Australia and New Zealand, a structured stakeholder dialogue combining evidence review and expert deliberation produced a regional SCI research strategy centered on collaborative networks, priority setting, and knowledge translation (42).

The Swiss strategy shares core features with these initiatives, including multi-stakeholder participation, prioritization process, and the integration of scientific evidence with lived experience. The priorities identified also show considerable convergence across countries, particularly regarding secondary conditions, psychosocial support, long-term follow-up, accessibility, and participation.

The Swiss strategy nevertheless differs from these initiatives in scope. Whereas previous initiatives have primarily focused on research agendas, the Swiss strategy attempts to translate shared priorities into a policy and implementation framework, with a governance structure, recurring implementation cycles, and monitoring mechanisms. In this respect, it contributes to operationalizing the vision of the WHO International Perspectives on Spinal Cord Injury report (2): moving from identifying what matters towards creating structures for coordinated action, clarification of responsibilities, and monitoring of progress. The transition from priority setting towards implementation remains relatively uncommon in the SCI field and provides an opportunity to examine the conditions, challenges, and adaptations required to sustain such efforts over time.

International comparisons also underline why several fields of action were selected. Recent Canadian work documented wide inequities in public funding for essential SCI-related supplies, services, and mobility equipment, with access varying by province and by injury cause (43). Comparable risks of fragmentation exist in Switzerland, where coverage differs between accident and health insurance schemes and across cantons (9). The fields of action on safeguarding existing services, mobility and accessibility, and lifelong medical follow-up respond directly to these system-level vulnerabilities.

### Implementation in a decentralized health system: challenges and enabling conditions

Implementing the Swiss National Strategy for SCI presents both opportunities and structural challenges. Unlike many national strategies, this initiative was developed in a bottom-up process rather than initiated by federal authorities (44, 45). Its implementation therefore depends on sustained engagement across federal, cantonal, institutional, and private actors. To support this, a broad stakeholder network was established from the outset, including clinicians, researchers, public authorities, insurers, and people with SCI, complemented by a national Forum to strengthen cross-sector collaboration and ownership.

A first challenge relates to Switzerland's federal structure and the resulting fragmentation of health service organization and financing across 26 cantons (9). Switzerland's federal governance system limits uniform implementation across cantons, requiring coordination rather than directive control. Moreover, as there is no automatic mechanism for national scale-up, implementation depends on coordination and voluntary alignment across cantonal systems, which may differ in priorities and resources.

A second challenge is the mismatch between short-term budgeting cycles and long-term system goals. Measures such as strengthening long-term follow-up or caregiver support require sustained and intersectoral investment, while funding decisions often prioritize immediate and measurable outcomes. In the absence of a dedicated national mechanism for evaluating and scaling rehabilitation innovations, promising initiatives may depend on the commitment of individual institutions, cantons, or insurers to achieve long-term sustainability.

A third challenge concerns implementation capacity. Health and social services are already facing workforce shortages and increasing demands (25, 27). New initiatives therefore need to be integrated into existing structures and workflows to avoid administrative burden and implementation fatigue.

A fourth challenge arises in areas requiring legislative or intersectoral action beyond the health system, such as accessibility regulation or financial protection mechanisms. These measures depend on political alignment across sectors and are therefore more difficult to advance within implementation cycles.

Despite these challenges, several factors provide a strong foundation for implementation. The SCI field in Switzerland benefits from longstanding collaboration between specialized centers, rehabilitation researchers, NGOs, and user organizations. These networks can support coordination even in the absence of formal mandates.

Another asset is Switzerland's established data infrastructure, particularly the SwiSCI cohort study (10, 11). By combining longitudinal clinical data from initial rehabilitation with self-reported data on functioning, quality of life, health conditions, participation, service use, and environmental factors, SwiSCI provides a valuable basis for identifying trends and monitoring system-level developments. Effective learning systems require clearly defined indicators, transparent monitoring processes, and mechanisms that connect evidence with decision-making (38, 39).

Recent national recommendations on quality monitoring and public reporting in Switzerland emphasize exactly that continuous improvement depends on structured use of data, stakeholder involvement and feedback mechanisms (46). The Swiss strategy builds on this principle by aiming to integrate monitoring and review processes into future implementation cycles, with the goal of using evidence to inform adaptation over time.

Implications for rehabilitation strategies in other contexts

The Swiss experience provides insights into how national rehabilitation strategies may be developed through structured participatory governance, even in decentralized systems. Several elements appear particularly important: stable coordination structures, involvement of people with lived experience, access to relevant data infrastructures, and sustained commitment from participating organizations.

This strategy development process may be particularly relevant for populations requiring long-term rehabilitation and support, where health outcomes are closely linked to functioning, participation, and environmental factors. However, applying similar approaches in other contexts would require adaptation to existing governance structures, financing mechanisms, and the capacity and willingness of relevant actors to engage in collective implementation.

### Limitations

The development process reflects a single-country experience embedded in a specific institutional and health-system context, which may limit transferability to other settings with different governance structures and resources. In addition, the participatory process relied on engagement from established stakeholders within existing SCI networks. Although efforts were made to include diverse perspectives, some groups may have been underrepresented.

As implementation has only recently started, this study does not yet evaluate the effectiveness or long-term impact of the strategy. Future work will be required to assess whether the governance model and implementation mechanisms successfully translate strategic priorities into measurable improvements in care, participation, and equity.

## Conclusion

The Swiss National Strategy for SCI demonstrates the feasibility of developing a shared strategic framework that integrates clinical, social, and policy perspectives across multiple stakeholder groups. The development process highlighted the importance of defining shared priorities among relevant stakeholders and establishing governance structures that can support subsequent implementation. As implementation progresses, additional work will be needed to strengthen institutional ownership, integrate selected measures into existing structures, and establish appropriate approaches for monitoring implementation progress.

For policy and practice, the experience highlights that, in decentralized systems without a single implementing authority, national strategies depend on sustained engagement and ownership from the organizations responsible for service delivery, financing, and coordination. The next phase will therefore depend on how participating organizations translate shared priorities into feasible actions within their existing mandates and resources.

## Data Availability

The information supporting the findings of this study is included in the article. Further inquiries can be directed to the corresponding author.
